# Comparative Analysis of the Expression Profile of *Wnk1* and *Wnk1/Hsn2* Splice Variants in Developing and Adult Mouse Tissues

**DOI:** 10.1371/journal.pone.0057807

**Published:** 2013-02-25

**Authors:** Masoud Shekarabi, Ron G. Lafrenière, Rébecca Gaudet, Janet Laganière, Martin M. Marcinkiewicz, Patrick A. Dion, Guy A. Rouleau

**Affiliations:** 1 Center of Excellence in Neuroscience of the Université de Montréal (CENUM), Centre de Recherche du Centre Hospitalier de l'Université de Montréal (CRCHUM), University of Montreal, Montreal, Quebec, Canada; 2 Cytochem Inc, Montreal, Quebec, Canada; 3 Department of Pathology and Cellular Biology, University of Montreal, Montreal, Québec, Canada; 4 CHU Sainte-Justine Research Center and Department of Paediatrics and Biochemistry, University of Montreal, Montreal, Quebec, Canada; 5 Department of Medicine, University of Montreal, Montreal, Québec, Canada; INSERM, France

## Abstract

The With No lysine (K) family of serine/threonine kinase (WNK) defines a small family of kinases with significant roles in ion homeostasis. WNK1 has been shown to have different isoforms due to what seems to be largely tissue specific splicing. Here, we used two distinct *in situ* hybridization riboprobes on developing and adult mouse tissues to make a comparative analysis of *Wnk1* and its sensory associated splice isoform, *Wnk1/Hsn2*. The hybridization signals in developing mouse tissues, which were prepared at embryonic day e10.5 and e12.5, revealed a homogenous expression profile with both probes. At e15.5 and in the newborn mouse, the two probes revealed different expression profiles with prominent signals in nervous system tissues and also other tissues such as kidney, thymus and testis. In adult mouse tissues, the two expression profiles appeared even more restricted to the nervous tissues, kidney, thymus and testis, with no detectable signal in the other tissues. Throughout the nervous system, sensory tissues, as well as in *Cornu Ammonis* 1 (CA1), CA2 and CA3 areas of the hippocampus, were strongly labeled with both probes. Hybridization signals were also strongly detected in Schwann and supporting satellite cells. Our results show that the expression profiles of *Wnk1* isoforms change during the development, and that the expression of the *Wnk1* splice variant containing the *Hsn2* exon is prominent during developing and in adult mouse tissues, suggesting its important role in the development and maintenance of the nervous system.

## Introduction

The With No K (lysine) kinase (WNK) family of serine/threonine protein kinases includes four members, WNK1 to WNK4, which are expressed in mammalian tissues. WNK kinases regulate the activities of ion channels and cotransporters by modulating their trafficking and surface expression, as well as by influencing their signaling pathways [1], [2]. Therefore, the WNKs indirectly regulate salt retention in the kidney and ion balance in nervous tissues, suggesting diverse functional roles in the organism. It is also proposed that the members of the WNK kinase family interact with each other and influence each other's function [2].

Tissue–specific distribution patterns of *WNK's* splice variants have been reported. A kidney specific, but kinase deficient variant of *Wnk1* was proposed to have a tissue–specific promoter displaying tissue–specific expression [3]. In addition, alternative skipping of exons 9, 11, 12, 26, 26a, 26b and *Hsn2* of *Wnk1* in a tissue–specific fashion and isoforms lacking exon 18 and 22 of *Wnk3* were also reported, but the physiological roles of these variants remain to be investigated [3]–[7].

The detailed expression profile of each *WNK* kinase is not fully determined. However, their expression appears to be ubiquitous in developing and adult rodents as well as in human tissues [6], [8]. *Wnk1* expression was shown to be high in adult mouse tissues such as heart, testis, and kidney by a BAC reporter assay *in vivo* [9]. RT-PCR analysis has also shown a predominant expression for *Wnk2* in heart, brain, and colon in human [8]. In addition, RT-PCR analysis of *Wnk3* splice variants in mouse has shown low expression of exon 18a containing isoforms in the adult mouse brain, kidney, liver, lung and pancreas, while isoforms with exon 18b are only detected in brain [6], [10]. *Wnk1* and *Wnk4* mRNA and protein are also highly expressed in secretory epithelia [7], [11], [12].

The *WNK*s are responsible for at least two disorders in humans. Large deletions in the first intron of *WNK1* leads to an elevation in *WNK1* expression causing an autosomal dominant disease called pseudohypoaldosteronism type II (PHA II; OMIM #145260) or Gordon's syndrome [13], [14]. However, *WNK4* missense mutations abolish the protein's inhibitory activity on the Na^+^/Cl^−^ cotransporter (NCC) and this leads to the same phenotype observed with the elevated expression of WNK1. Patients with PHA II show increased sodium retention, hypertension and hyperkalemia. In addition, our group recently reported mutations in a *WNK1* isoform deemed to contain a nervous system–specific exon (formally referred to as the *HSN2* gene in hereditary sensory and autonomic neuropathy type II (HSANII; OMIM #201300) disorder [4]. HSAN II is associated with loss of sensation to pain, heat, and touch in distal areas of limbs, but no hypertension associated with pseudohypoaldosteronism has ever been reported in these patients. We previously noted that Wnk1/Hsn2 protein and mRNA were expressed in the nervous tissues of adult mice; expression was substantially stronger in dorsal root ganglia, satellite, and Schwann cells of the peripheral nervous system (PNS) [4]. Our results confirmed that *Wnk1* is ubiquitously expressed in all mouse tissues, with a higher expression in kidney and testis, but the *Wnk1/Hsn2* splice variant was proposed to be predominantly expressed in nervous tissues from the adult mouse [4].

Although the expression of *Wnk1* and *Wnk1/Hsn2* isoforms were predominantly detected in tissues of the cardiovascular and nervous systems of the adult mouse, respectively, their expression profiles in most adult mouse tissues during mouse development remained unknown. In the present report, we used *in situ* hybridization (ISH) to explore and compare the expression profile of the *Wnk1* and *Wnk1/Hsn2* mRNA isoforms in developing and adult mouse tissues. We used a mouse specific *Wnk1* riboprobe which hybridizes with all known *Wnk1* mRNA isoforms, including nervous tissues and kidney specific variants. However, our *Wnk1/Hsn2* riboprobe hybridizes only to the *Hsn2* exon containing isoforms. Our results revealed that the *Wnk1* and *Wnk1/Hsn2* mRNA isoforms expression patterns change as development advances. Furthermore, we show that *Wnk1* and *Wnk1/Hsn2* mRNA isoforms are ubiquitously detected in the embryonic days e10.5 and e12.5, and heterogeneously in e15.5, newborn (p1), postnatal (p10), and adult mouse stages. The distribution patterns of the mRNAs suggest spatial overlapping with regional differences in mRNA transcript concentrations. Predominant *Wnk1* mRNA expression was noted in kidney, brain, olfactory neuroepithelium, thymus and spleen of the adult mouse.Moreover, *Wnk1* mRNA isoforms' distribution pattern was noted in the heart, testis, cranial and spinal ganglia with predominant *Wnk1/Hsn2* mRNA expression in nervous tissues, testis, and kidney.

## Materials and Methods

### Ethics Statement

Animal care, euthanasia and necropsy assays were performed in accordance with guidelines of the Canadian Council on Animal Care. The protocol for the frozen tissue bank of embryonic, postnatal and adult rat and mouse tissues was approved on October 25, 2007 and re-approved on March 20, 2012 by Cytochem's internal Ethics Committee presided by Dr. Marius Gangal, MD, PhD.'

### cRNA probe preparation

*Wnk1* and *Wnk1/Hsn2* radioactive cRNA probes were prepared from cloned RT-PCR fragments of 805 bp and 435 bp, previously amplified from mouse brain cDNA, with specific activities of approximately 1,060 and 640 Ci/mmol, respectively. All RT-PCR was performed as previously described (Shekarabi et al., 2008) except that in the present report, Superscript III (Invitrogen) was used to synthesize the cDNA using the following primers: for *Wnk1*: 5′-TGACATCGAAATCGGCAGAGGCT-3′ and 5′-GGGTACGGGTAGAATTAGCAGAAG-3′; for the *Wnk1/Hsn2* specific probe: 5′-CAAGGAACCACATCTCAGCAGGTCT-3′ and 5′-CATACTGTTCTGCCACCCGGGCCTGGTAATG-3′; for *KS-Wnk1* amplifications 5′- CTCATTGCTGCTGCTGTTCTC -3′ (forward, exon 4A specific) and 5′- GAAACTGGACGTTCATGAATAG -3′ (reverse, exon Hsn2 specific) were used. The Wnk1 and Wnk1/Hsn2 fragments were then cloned into pGEM-9Zf (–) Vector (Promega) with the SP6 or T7 promoter on either side, respectively. The Wnk1 template spans from the 3′ end of exon one to the 3′ end of exon 6 of *Wnk1* (accession number: NM_001199083.1, 1693–2497 bp) (Shekarabi et al., 2008) and the Wnk1/Hsn2 template was amplified from the 5′ end of the *Hsn2* exon to a Bgl I site in its ORF (4192–4626 bp). cRNAs were then produced *in vitro* from the DNA templates after linearization with Xba I or Sal I in the presence of T7 or SP6 RNA polymerase enzymes, generating sense or antisense products, respectively. The assay proved that the DNA templates were functional and, hence, were used to synthesize the radioactive probes. Both sense and antisense radio-labeled riboprobes were synthesized according to the manufacturer's specifications (Ambion) and labeled with ^35^S-UTP (>1,000 Ci/mmol; Cat. # NEG039H, PerkinElmer LAS Canada, Inc.).

### Tissue preparation and in Situ Hybridization

The tissue preparation was essentially performed as described previously [15]. Tissues examined in this assay included whole-body sections of embryonic, newborn, postnatal and adult mice. The tissues were frozen in isopentane, cooled to −35°C and conserved at −80°C in tightly-closed plastic bags. Then, 10 µm sections were cut on a cryostat and mounted on glass microscope slides. The sections were treated with triethanolamine/acetic anhydride, washed and dehydrated through a gradient of ethanol solutions. The sections were then hybridized with ^35^SUTP -labeled cRNA antisense and sense probes generating positive and negative (control) signals, respectively. Subsequently, sections were hybridized overnight at 55°C in 50% deionized formamide, 0.3 M NaCl, 20 mM Tris-HCl, pH 7.4, 5 mM EDTA, 10 mM NaPO4, 10% dextran sulfate, 1×Denhardt's, 50 µg/ml total yeast RNA, and a 50–80,000 cpm/µl ^35^S-labeled cRNA probe. The tissues were subjected to stringent washing at 65°C in 50% formamide, 2×SSC (saline-sodium citrate, 20×stock solution consists of 3 M sodium chloride and 300 mM tri-sodium citrate which is adjusted to pH 7.0 with HCl), and 10 mM DTT (dithiothreitol), followed by washing with PBS (phosphate buffered saline) before treatment with 20 µg/ml RNAse A at 37°C for 30 minutes. After washes in 2×SSC and 0.1×SSC for 10 minutes at 37°C, the slides were dehydrated, exposed to X-ray film for 4 days, then dipped in Kodak NTB nuclear track emulsion. Gene expression patterns were analyzed by both x-ray film autoradiography (3- or 4-day exposure time) and emulsion autoradiography (6- to 12-day exposure time) in light-proof boxes containing desiccant at 4°C. Prior to our *Wnk1* and *Wnk1/Hsn2* ISH experiments, the mouse tissues used here were all validated using riboprobes of Low-Density Lipoprotein (LDL) receptor mRNA.

### Immunohistochemistry

The immunohistochemistry experiments were performed using co-labeling with anti-Wnk1/Hsn2 (1∶4000) on 10 µm frozen sections of e10.5 embryos as previously described[4] and using anti-chicken Nestin (Neuromics; 1∶400) antibody or fluorescein-lycopersicon esculentum lectin (tomato lectin, Vector; 1∶1000); on 7 µm frozen sections of the adult mouse kidney using anti-mouse parvalbumin (Sigma; 1∶250) and on 10 µm frozen sections of the adult mouse trigeminal ganglion using anti-glutamate synthetase (Chemicon; 1∶150) antibodies. Alexa Fluor 555 secondary anti-rabbit, Alexa Fluor 488 secondary anti-mouse and Alexa Fluor 488 secondary anti-chicken antibodies (Molecular Probes; Invitrogen), respectively, were used (1∶1,000) to visualize rabbit, mouse and chicken primary antibodies. The slides were then analyzed as described previously[4].

### Imaging

Photographic development of emulsion autoradiography was carried out at 17°C using Kodak D-19 developer solution diluted 1∶1 with water and Kodak fixer (Cat # 1971746) as indicated by the manufacturer. After development, the slides were air-dried in a dust-free environment and then lightly counterstained with cresyl violet to visualize cell nuclei. The results were analyzed under a Reichert Polyvar microscope equipped with Darklite and Nomarski attachments, under both bright- and dark-field illumination. All images were adjusted as to brightness and contrast to clearly display their details. Hybridization with antisense cRNA probes revealed silver labeling within the sites of gene expression, whereas control hybridization with sense cRNA probes displayed non-specific labeling at the background level.

## Results

### *Wnk1* and *Wnk1/Hsn2* isoforms are expressed in developing mouse tissues

*Wnk1* knockout mice die of cardiovascular abnormalities before embryonic day 13 (e13) [16]. This underscores the crucial role of embryonic *Wnk1* expression during development. To test the expression profiles of *Wnk1* and *Wnk1/Hsn2* mRNA isoforms in developing and adult mouse tissues, we employed the *in situ* hybridization (ISH) technique. To prepare the vector encoding the sense and anti-sense *Wnk1* ripoprobes, we used a mouse brain cDNA library and oligonucleotide primers that were designed to PCR amplify the 805 bp region located between the end of exon 1 to exon 6 of *Wnk1*. For the vector encoding the *Wnk1/Hsn2* cRNA probes, we used a 435 base pair fragment derived from the 5′ end to the Bgl I site in *Hsn2* exon. The integrity and size of the templates, as well as the riboprobes, were confirmed on a DNA agarose gel using non-radioactive probes (data not shown). The specificity of these probes was previously shown by our group using Northern blot experiments [4].

**Figure 1:**
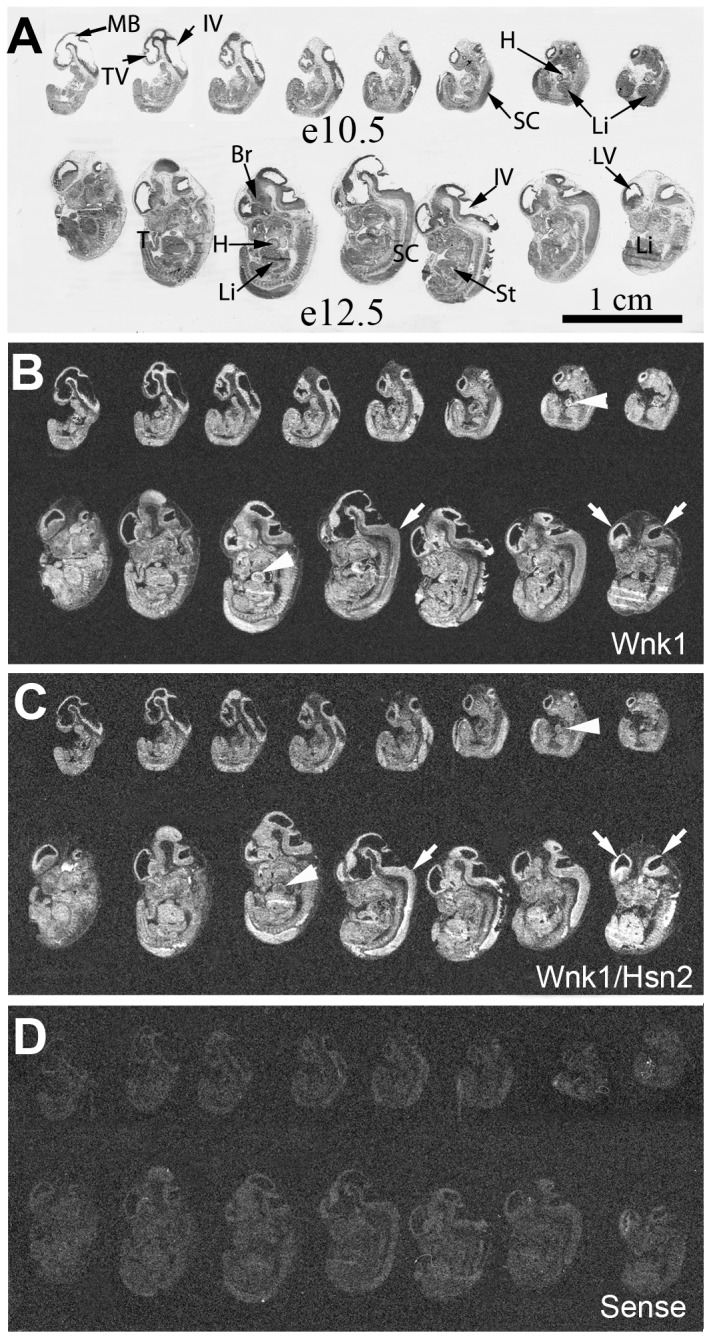
*Wnk1* and *Wnk1/Hsn2* expression in e10.5 and e12 day old embryos. Whole body sections of mouse embryos at e10.5 and e12.5 days of embryonic development were used in ISH detections with specific riboprobes. (A) Cresyl violet stained body sections of sections hybridized with the riboprobes in the panels below. (B–D) Sections respectively hybridized with anti-sense *Wnk1*, anti-sense *Wnk1/Hsn2*, sense *Wnk1/Hsn2* (negative control) riboprobes. Arrows in B and C respectively indicate the *Wnk1* and *Wnk1/Hsn2* signals in lateral brain ventricles and spinal cord. Arrowheads in B and C respectively indicate the increased *Wnk1* signal, in comparison to the *Wnk1/Hsn2* signal in the heart. IV  =  4^th^ ventricle; Br  =  brain; H  =  heart; Li  =  liver; LV  =  lateral ventricle; MB  =  midbrain; SC  =  spinal cord; St  =  stomach; T  =  tail; TV  =  telencephalic vesicle.

Parallel to the ISH detections, we prepared reference sections using cresyl violet on cuts adjacent to those hybridized (Fig. 1A). We then used the *Wnk1* and *Wnk1/Hsn2* anti-sense riboprobes independently (Fig. 1B, 1C); a *Wnk1/Hsn2* sense riboprobe was used as a negative control probe (Fig. 1D). At both e10.5 and e12.5, a ubiquitous hybridization signal was observed for *Wnk1* and *Wnk1/Hsn2*, presumably because of the developing vasculature and nervous system. To confirm the expression of Wnk1/Hsn2 in these regions, the Wnk1/Hsn2 antibody and fluorescein-lycopersicon esculentum lectin (tomato lectin) were used to co-label endothelial cells lining blood vessels. While the vasculature appeared to express Wnk1/Hsn2 at this stage, many areas remained strongly Wnk1/Hsn2 positive and excluded tomato lectin labeling. To further identify the excluded Wnk1/Hsn2 positive cells, we co-immunostained the sections with anti-Wnk1/Hsn2 and anti-Nestin antibodies. These later immunohistochemistry experiments revealed that neuronal crest cells and proliferative endothelial cells are strongly positive for Wnk1/Hsn2 antibody, suggesting the expression of this splice variant in the developing nervous system (Fig. S1).

At both stages, e10.5 and e12.5, *Wnk1* expression was evident in the lateral brain ventricle and spinal cord, an observation suggestive of a contribution to nervous system development (Fig. 1B, 1C; Table 1). From e10.5 to e12.5, the expression of *Wnk1/Hsn2* appeared to become stronger in the spinal cord in comparison to *Wnk1*. In fact, it seemed that the expression of *Wnk1/Hsn2* was greater in that specific area than the rest of the developing embryo. Another difference between *Wnk1* and *Wnk1/Hsn2* was visible at e10.5 as the *Wnk1* hybridization signals appeared to be stronger than the *Wnk1/Hsn2* signal in the developing heart (Fig. 1B, arrowheads). However, this observation is consistent with the cardiovascular developmental defects in the *Wnk1* null mouse at this age, with death before e13.5 [16]. A stronger *Wnk1* signal could possibly be detected because of the longer size of the *Wnk1* probe. In all e12.5 developing tissues a low to moderate, but ubiquitous, mRNA expression profile was observed for both *Wnk1* and *Wnk1/Hsn2*.

**Table 1:** Semi-quantitative evaluation of *Wnk1* and *Wnk1/Hsn2* expression from e10.5, e12.5, e15.5 and p1 mice embryo whole-body sections.

Stage	Tissue	*Wnk1*	*Wnk1/Hnk2*		Comment	
e10.5	brain	+++		++		ubiquitousm RNA distribution
	spinal cord	+++		++		
	spinal ganglia	+++		+		
	heart	+++		+		
	liver	+++		+		
e12.5	brain	++		++		ubiquitous mRNA distribution
	spinal cord	++		++++		
	spinal ganglia	++		++		
	heart	+++		+		
	liver	+++		++		
e15.5	brain	++		++		ubiquitous mRNA distribution
	spinal cord	++		++++		
	spinal ganglia	++		+++		
	heart	++		+		
	liver	++		+++		
p1	olfactory neuroepithelium	++++		++++		heterogeneousm RNA distribution
	brain	++		+++		
	spinal cord	+		+++		
	spinal ganglia	++		+++		
	trigeminal ganglia	+++		++		
	lung	+++		+		
	heart	+++		+		
	Liver	+		+		
	kidney	+++		+++		
	thymus	++++		+++		
	spleen	NE		NE		
	testis	NE		NE		

Average labeling level: +  =  very weak; ++  =  weak; +++  =  medium; ++++  =  high mRNA concentration.

**Figure 2:**
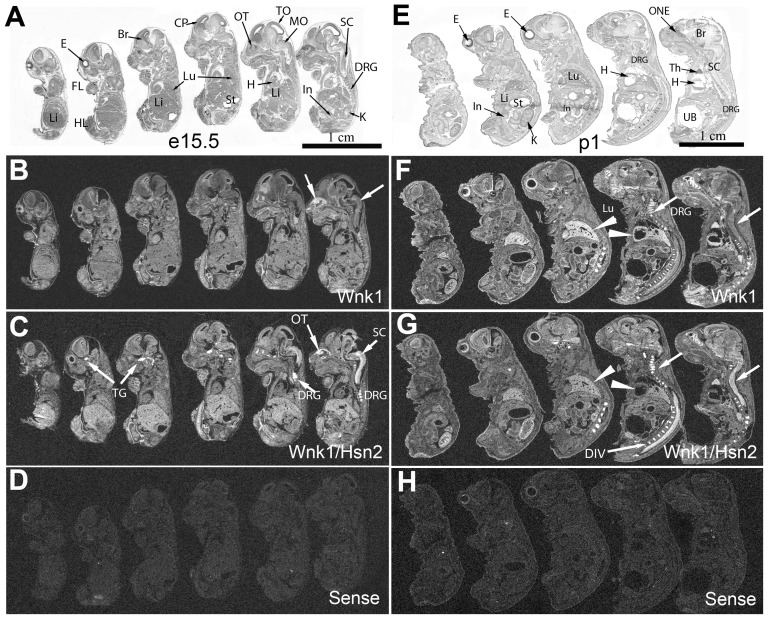
*Wnk1* and *Wnk1/Hsn2* expression in e15.5 embryos and newborn (p1) mice. Whole body sections of mouse embryos at e15.5 days and p1 of embryonic development were used in ISH detections with specific riboprobes. (A, E) Whole body sections of e15.5 and p1 mouse were stained with cresyl violet. (B, F; C, G; and D, H) Sections respectively hybridized with anti-sense *Wnk1*, anti-sense *Wnk1/Hsn2*, and sense *Wnk1/Hsn2* (negative control) riboprobes. Arrows in B indicate the spinal cord and olfactory neuroepithelium. Arrows in C and G indicate the increased *Wnk1/Hsn2* signal detected in the nervous tissues and intervertebral discs. Arrowheads in F and G indicate the increased *Wnk1* signals in the heart and lung. Br  =  brain; CP  =  cortical plate; DIV  =  intervertebral disc; DRG  =  dorsal root ganglion; E  =  eye; FL  =  forelimb; H  =  heart; HL  =  hindlimb; In  =  intestine; K  =  kidney; Li  =  liver; Lu  =  lung; MO  =  medulla oblongata; ONE  =  olfactory neuroepithelium; OT  =  olfactory turbinates; SC  =  spinal cord; St  =  stomach; TG  =  trigeminal ganglion; TO  =  tectum opticum; Th  =  thymus; UB  =  urinary bladder.

We used the same riboprobes to examine expression in the e15.5 and postnatal one (p1) sagittal sections at later embryonic stages (Fig. 2). At e15.5, hybridization with the *Wnk1/Hsn2* riboprobe revealed a stronger signal than the *Wnk1* riboprobe in nervous system tissues; the highest expression being in sensory tissues such as dorsal root ganglia (DRG), spinal cord (SC), trigeminal ganglia (TG) and olfactory turbinates (OT) (Fig. 2B, 2C, arrows). As for the e10.5 and e12.5 stages, a relatively uniform expression profile for both probes was still observed in all tissues at both e15.5 and p1. However, a possibly stronger *Wnk1* signal was visible in the developing lung and heart at p1 (Fig. 2F, 2G, arrowheads). In p1 animals, the expression of *Wnk1/Hsn2* was also increased in the olfactory neuroepithelium, brain, spinal cord, DRG, kidney and thymus (Fig. 2G, Table 1). Overall, at both e15.5 and p1, the brain and the spinal cord displayed more intense signal for *Wnk1/Hsn2* than *Wnk1*. Intervertebral discs were also strongly labeled with both riboprobes, but a stronger signal was detected with the *Wnk1/Hsn2* probe (Fig. 2G). No hybridization signals were detected when the *Wnk1/Hsn2* sense probe was used (Fig. 2D, 2H).

**Figure 3:**
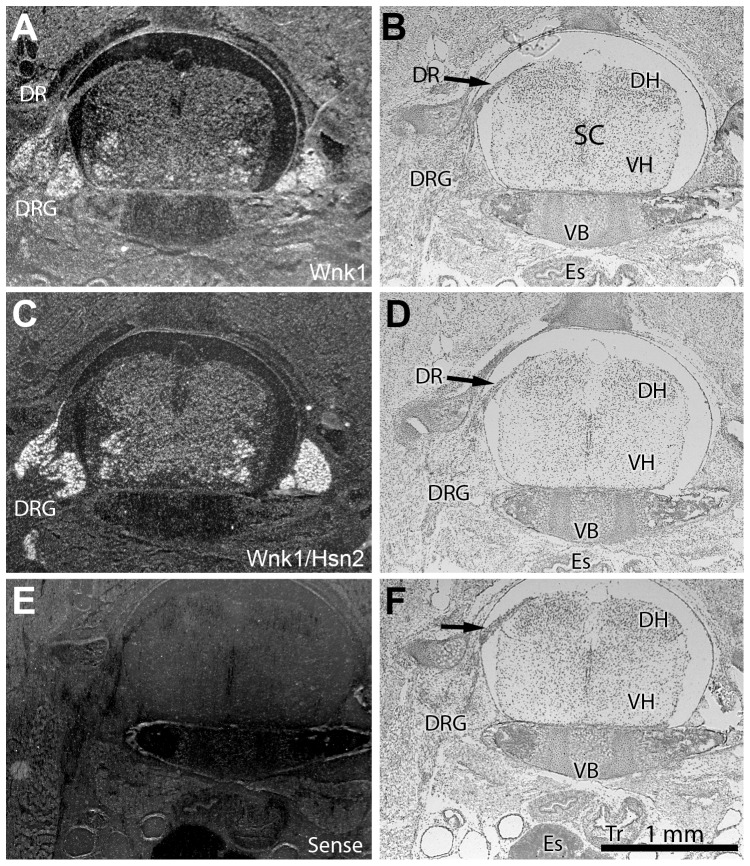
*Wnk1* and *Wnk1/Hsn2* expression in the cervical spinal cord of p1 mice. (A, C and E) Cross sections from the cervical spinal cord of p1 mice were respectively used in ISH detections with anti-sense *Wnk1*, anti-sense *Wnk1/Hsn2* and sense *Wnk1/Hsn2* (negative control) ribobrobes. (B, D and F) Matching cross sections were stained with cresyl violet. (E) Hybridization with the sense cRNA probe recognizing *Wnk1/Hsn2* did not show any signal. DH  =  dorsal horn; DR  =  dorsal root; DRG  =  dorsal root ganglion; Es  =  esophagus; SC  =  spinal cord; Tr  =  trachea; V  =  ventral horn; VB  =  vertebrae body.

**Figure 4:**
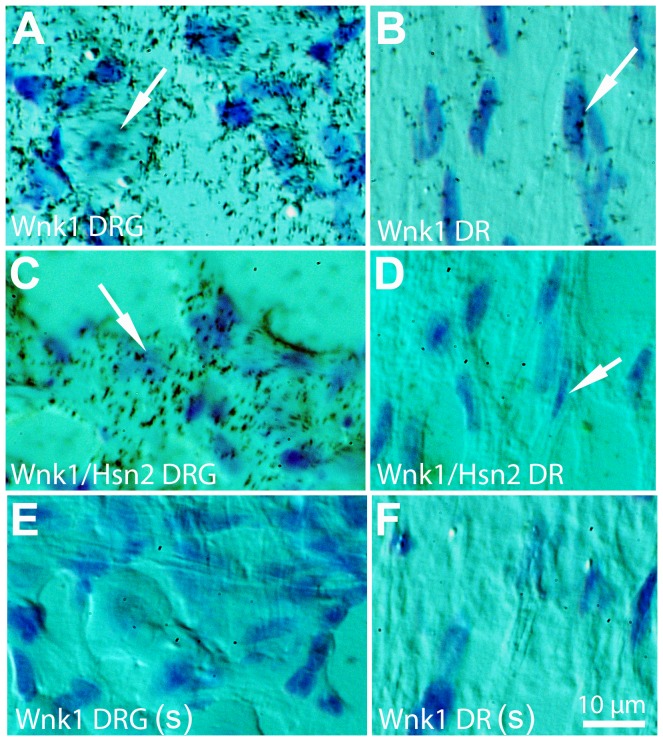
*Wnk1* and *Wnk1/Hsn2* expression in the DRGs of adult mice. DRG (A, C, and E) and dorsal root (B, D, and F) sections of adult mice were used in ISH detections using anti-sense *Wnk1*, anti-sense *Wnk1/Hsn2* and sense *Wnk1/Hsn2* (negative control) riboprobes. Emulsion autoradiographies revealed intense *Wnk1* and *Wnk1/Hsn2* signals in the neuronal somata and in DR (dorsal root) areas (arrows in A–D). (E and F) No hybridation signal was detected with the sense *Wnk1/Hsn2* probe. DRG  =  dorsal root ganglia; DR  =  dorsal root.

**Figure 5:**
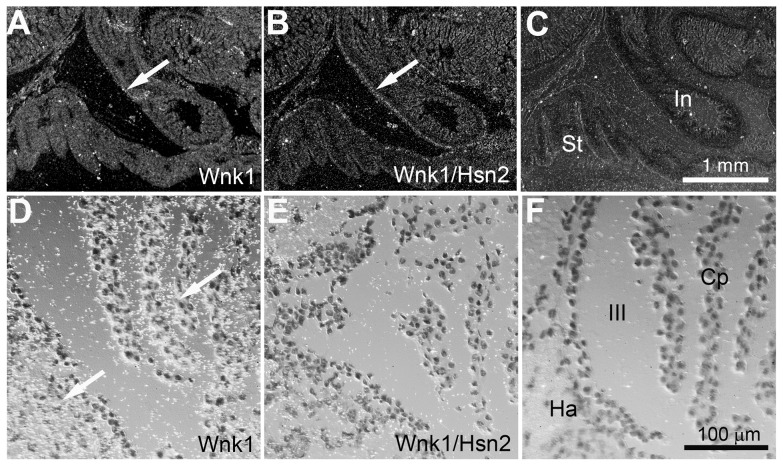
*Wnk1* and *Wnk1/Hsn2* expression in the myenteric plexus of p1 mice and the choroid plexus and habenula of the adult mouse. (A–C) Neuronal ganglia of the myenteric plexus (mice at p1 stage) were examined in ISH detections with riboprobes against anti-sense *Wnk1*, anti-sense *Wnk1/Hsn2* and sense *Wnk1/Hsn2* (negative control) riboprobes. X-ray autoradiography of the plexus showed a weaker signal for the *Wnk1* riboprobe (arrow in A) than the one for *Wnk1/Hsn2* (arrow in B). Arrows in A and B indicate the intestine wall musculature. (D–E) Further examination using emulsion autoradiography revealed strong *Wnk1* labeling in the choroid plexus and habenula of the adult mouse (arrows in D). (F) The sense *Wnk1/Hsn2* riboprobe did not display any signal in a corresponding section. III  =  the third ventricle; CP  =  choroid plexus; Ha  =  habenula; In  =  intestine; St  =  stomach.

In spinal cord transverse sections prepared from p1 animals, both *Wnk1* and *Wnk1/Hsn2* signals could be observed in the ventral, ventromedial, medial nuclei and DRGs (Fig. 3A, 3C). However, the *Wnk1/Hsn2* signal appeared stronger. The early expression of Wnk1 in the dorsal roots suggests that Wnk1 might also be important for the development of sensory roots. The DRG neurons and dorsal root, which consist of nerve roots connecting the spinal ganglia with the spinal cord, showed hybridization signals for both *Wnk1* and *Wnk1/Hsn2* in the adult mouse (Fig. 4A–F). In the enteric nervous system, the myenteric plexus, which includes a few neuronal ganglia (seen within the intestinal smooth muscle layer), displayed much less intense *Wnk1* labeling than *Wnk1/Hsn2* at p1 (Fig. 5A, 5B, respectively). A possibly stronger *Wnk1* labeling was also detected in the choroid plexus of the third ventricle, as well as the habenula commissure (Fig. 5D, 5E). This finding is consistent with the high expression of the potassium/chloride cotransporter 3 (KCC3), a phosphorylation substrate of Wnk1, in the choroid plexus [17], [18].

### Expression of *Wnk1* and *Wnk/Hsn2* mRNA isoforms in postnatal and adult mouse tissues

**Figure 6:**
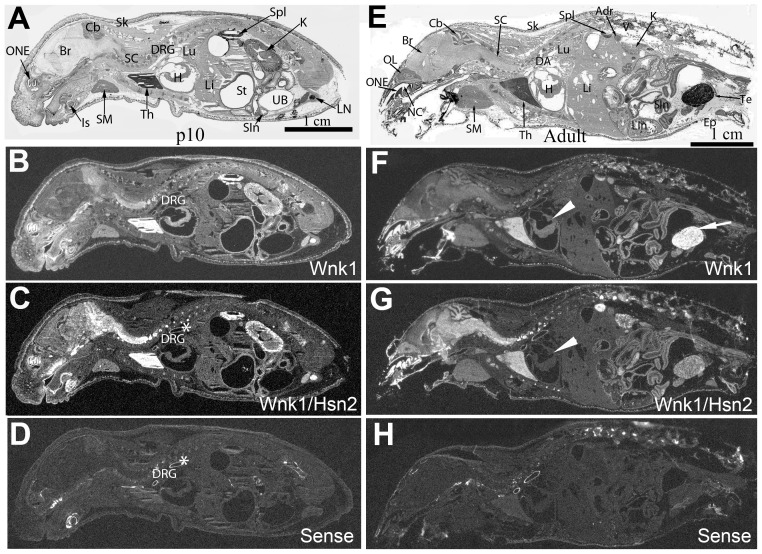
*Wnk1* and *Wnk1/Hsn2* expression of isoforms in p10 and adult mice. Whole body sections were used in ISH detections with specific riboprobes. (B, F; C, G, and D, H) Whole-body sagittal sections of p10 (A–D) and the adult mouse (E–H) were respectively hybridized with anti-sense *Wnk1* (B and F), anti-sense *Wnk1/Hsn2* (C and G) and sense (negative control) riboprobes (D and H). Corresponding sections were also stained with cresyl violet (A and E). At these stages, most of the hybridized signal was observed in the nervous tissues, testis, kidney, and spleen. The heart showed greater *Wnk1* labeling than *Wnk1/Hsn2* (arrowheads in F and G). The asterisks (*) indicate nonspecific labeling in the dorsal aorta. Adr  =  adrenal gland; Br  =  brain; Cb  =  cerebellum; DA  =  dorsal aorta; DRG  =  dorsal root ganglion; Ep  =  epididymis; H  =  heart; K  =  kidney; Is  =  incisor; Li  =  liver; Lin  =  large intestine; LN  =  lymph node; Lu  =  lung; NC  =  nasal cavity; OL  =  olfactory lobe; ONE  =  olfactory neuroepithelium; SC  =  spinal cord; Sin  =  small intestine; Sk  =  skin; SM  =  submaxillary gland; Spl  =  spleen; St  =  stomach; Te  =  testis; Th  =  thymus; UB  =  urinary bladder; V  =  vertebrae.

**Figure 7:**
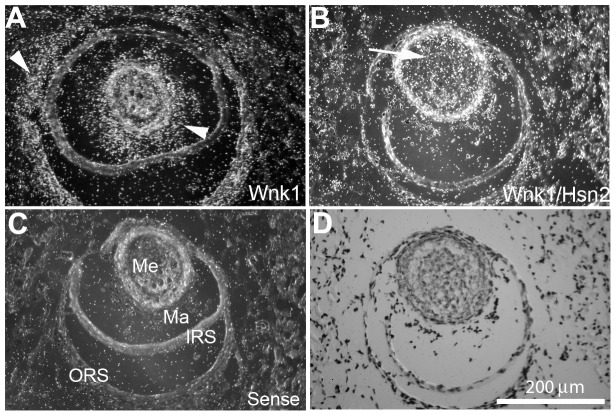
*Wnk1* and *Wnk1/Hsn2* expression in p10 mice vibrissae. (A, and B) Cross sections of sensory vibrissae (hair follicles) of p10 mouse mice were prepared and used in ISH detections with anti-sense *Wnk1* and *Wnk1/Hsn2* riboprobes, respectively. (C) A negative control hybridization was prepared using the sense *Wnk1/Hsn2* riboprobe. Arrowheads in A indicate increased *Wnk1* signal in the vibrissa matrix and outer root sheath. The arrow in B indicates an increased *Wnk1/Hsn2* signal in the medulla. (D) A corresponding section was stained with cresyl violet. IRS  =  inner root sheath; Ma  =  matrix; Me  =  medulla; ORS  =  outer root sheath.

At later postnatal stages, the mRNA expression of both *Wnk1* and *Wnk1/Hsn2* continued to become even more restricted to certain tissues. At p10, *Wnk1* mRNA expression displayed a similar distribution to the one observed earlier (p1) with more prominent expression in the kidney and immune system (thymus, spleen and lymph nodes); albeit the distribution of expression at p10 does not become drastically different in adult mice (Fig. 6B, 6F; Tables 2, 3). The heart showed a possible prevalent *Wnk1* labeling and very weak *Wnk1/Hsn2* mRNA at stage p10 with almost no *Wnk1/Hsn2* mRNA signal in the adult heart (Fig. 6F, 6G, arrowheads). In adult mice, most of the other tissues had signals of almost equal intensity for both riboprobes, except for testis where the *Wnk1* probe appeared to be stronger (Fig. 6F, arrow). Vibrissae are sensory organs in the mouse and they are the center for odor detection. A strong, but distinct pattern of distribution of *Wnk1* and *Wnk1/Hsn2* mRNAs was observed in p10 vibrissae (Fig. 7A, 7B). A much stronger *Wnk1/Hsn2* isoform was detected in the medulla when compared with the *Wnk1* signal which appeared to be stronger in the matrix (Fig. 7; Table 4).

**Table 2:** Semi-quantitative evaluation of *Wnk1* and *Wnk1/Hsn2* expression in p10 and p56 mice whole body sections.

Stage	Tissue	*Wnk1*	*Wnk1/Hnk2*		Comment	
p10	olfactory Neuroepithelium	++++		+++		heterogeneous mRNA distribution
	brain	++		++++		
	spinal cord	+++		++++		
	spinal ganglia	+++		+++		
	trigeminal ganglia	+		−		
	lung	++		−		
	heart	+++		+		
	Liver	++		+		
	kidney	++++		+++		
	thymus	++++		++++		
	spleen	+++		+++		
	testis	NE		NE		
Adult	olfactory Neuroepithelium	+++++		++++		heterogeneous mRNA distribution
	brain	++		++++		
	spinal cord	+		+++		
	spinal ganglia	++		+++		
	trigeminal ganglia	++		+++		
	lung	+		−		
	heart	++		−		
	Liver	+		−		
	kidney	+++		++		
	thymus	++++		+++		
	spleen	+++		++		
	testis	++++		++		

Average labeling level: +  =  very weak; ++  =  weak; +++  =  medium; ++++  =  high mRNA concentration. NE  =  not examined.

**Table 3:** Semi-quantitative evaluation of *Wnk1* and *Wnk1/Hsn2* expression in a 31-tissue array prepared from adult mice.

#	Tissue	*Wnk1*	*Wnk1/Hsn2*	Comments
1	brain	+++	+++	neurons
2	trigeminal	++	+++++	neurons
3	spinal cord	++	++	
4	heart	+++	−	
5	adneral gland	+++	+	
6	eye retina	++	++	
7	hypophysis	+++	++	
8	oviducts	++	+	
9	ovary	+++	++	
10	duodenum	+++	++	
11	ileum	++	+	
12	jejunum	++	+	
13	colon	+++	++	
14	uterus	+	−	
15	kidney	+++	++	distal convoluted tubes
16	liver	+	+	
17	gallbladder	+	+	
18	tongue	++	+	
19	thymus	++++	+++	
20	testis	++++	++	
21	lymph node	+++	+++	
22	submaxillary gland	+++	+	
23	urinary bladder	++	+	
24	spleen	++++	+++	
25	seminal	+	−	
26	lung	++	+	
27	stomach	++	−	
28	thyroid gland	+	−	
29	parathyroid gland	+	−	
30	mammary gland	++	+	

Average labeling level: +  =  very weak; ++  =  weak; +++  =  medium; ++++  =  high mRNA concentration

**Table 4:** Semi-quantitative evaluation of *Wnk1* and *Wnk1/Hsn2* expression in the vibrissae of p1 mice.

Vibrissae regions	*Wnk1*	*Wnk1/Hsn2*
medulla	+	+++
matrix	++++	+
inner root sheath	+	−
outer root sheath	+++	−

Score: −  =  not detectable; +  =  weak; ++  =  moderate; +++  =  medium; ++++  =  strong hybridization labeling.

**Figure 8:**
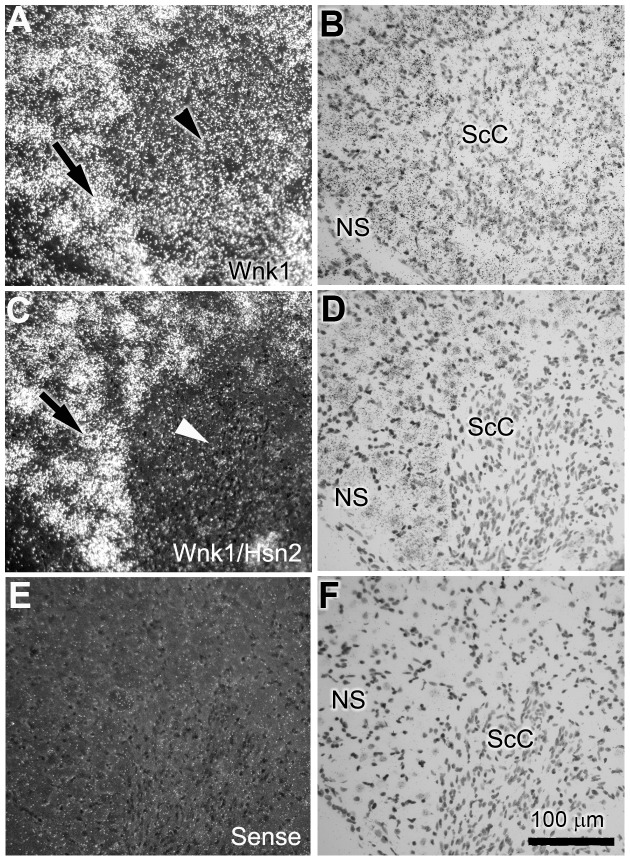
*Wnk1* and *Wnk1/Hsn2* expression in the DRG and ventral roots of p10 mice. (A, C and E) Cross sections of postnatal mouse DRG with exposed ventral root were respectively used in ISH detections with anti-sense *Wnk1*, anti-sense *Wnk1/Hsn2* and sense *Wnk1/Hsn2* (negative control) riboprobes. (B, D and F) Corresponding sections were stained with cresyl violet. Arrows in A and C indicate the signals from both probes in neuronal somata. Arrowheads in A and C identify the motor root region signal which shows increased *Wnk1* expression in comparison to the *Wnk1/Hsn2* expression. NS  =  neuronal somata; SC  =  Schwann cells.

**Figure 9:**
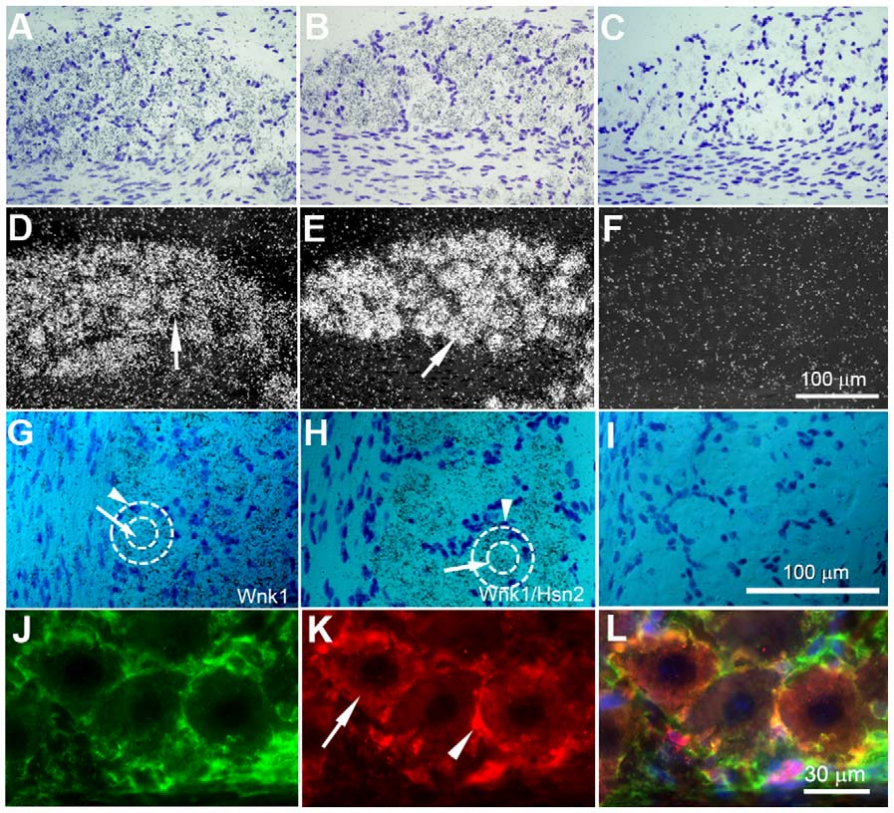
*Wnk1* and *Wnk1/Hsn2* expression in the peripheral nervous system of adult mice. (A–C) Tissue preparations from adult mouse trigeminal ganglion were stained with cresyl violet. (D–I) Corresponding sections were used in ISH detections with anti-sense *Wnk1*, anti-sense *Wnk1/Hsn2* and sense *Wnk1/Hsn2* (negative control) riboprobes; hybridization signals were detected by emulsion autoradiographies. Some sections were also co-immunolabeled (J–L) using anti-Wnk1/Hsn2 (red) and anti-glutamine synthetase (green) antibodies. Arrows in D and E show the expression of the *Wnk1* and *Wnk1/Hsn2* probes in trigeminal neurons. Arrows in G and H indicate the expression of *Wnk1* and *Wnk1/Hsn2*, respectively, and in K shows Wnk1/Hsn2 expression in the neuronal somata. Arrowheads in G and H point to the high labeling of the *Wnk1/Hsn2* riboprobe, and in K shows the high expression of Wnk1/Hsn2 in the supporting satellite cells. Dotted circles in G and H delineate the boundaries of neuronal somata and their supporting satellite cells.

The presence of *Wnk1* mRNA signals in the DRGs prompted us to examine peripheral nervous tissues in more detail. Consistent with our previous findings using immunohistochemistry and the anti-HSN2 antibody in adult mouse DRGs [4], we observed *Wnk1* labeling in Schwann cells of postnatal p10 mice of spinal motor nerves that traverse dorsal ganglia (Fig. 8A, arrowhead). Much less *Wnk1/Hsn2* mRNA expression was found in the corresponding nerve region (Fig. 8C, arrowhead). Overall, the *Wnk1* probe displayed a more evenly distributed signal in the nerve region, but the *Wnk1/Hsn2* signal predominated in spinal ganglia neuronal cells (Fig. 8C, arrow). Signals were detected inside as well as around the somata of trigeminal and DRG neurons which we confirmed to be from the supporting satellite cells (Fig. 9A–L and Fig. S2).

**Figure 10:**
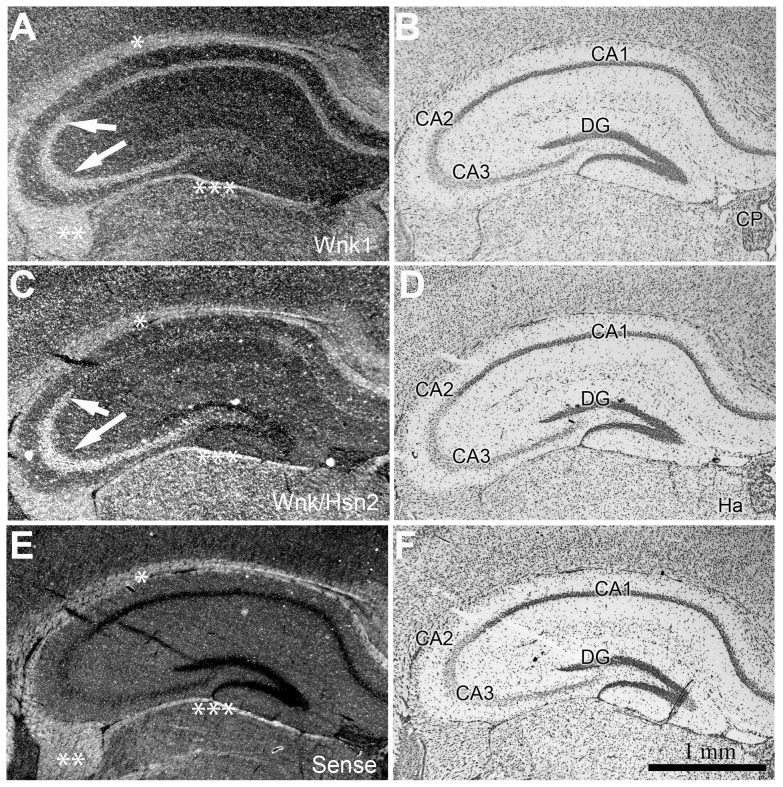
*Wnk1* and *Wnk1/Hsn2* expression in the hippocampus of adult mice. (A–F) Coronal sections of adult mouse hippocampus were examined using ISH detection with anti-sense *Wnk1*, anti-sense *Wnk1/Hsn2* and sense *Wnk1/Hsn2* (negative control) riboprobes, respectively. (B, D, and F) Corresponding sections were stained with cresyl violet. Arrows in A and C indicate increased signal in the CA2 and CA3 hippocampal areas with both probes. Asterisks indicate non-specific labeling in the corpus callosum (*), fimbria (**) and limitans (***).CA1–CA3  =  *Cornu Ammonis* area 1 to 3; DG  =  dentate gyrus; Ha  =  habenula; CP  =  choroid plexus.

Both the *Wnk1* and *Wnk1/Hsn2* probes revealed signals in the brain from early embryonic stages. In adult mice, the expression of both *Wnk1* and *Wnk1/Hsn2* appeared to be more restricted, as it was essentially seen in neurons of the adult mouse hippocampus (*Cornu Ammonis* 1, CA2, and CA3 areas) (Fig. 10A, 10C). The *Wnk1* and *Wnk1/Hsn2* patterns of mRNA expression in the hippocampus of adult mice brain appeared very similar to the one previously reported for *Wnk3* [10].

**Figure 11:**
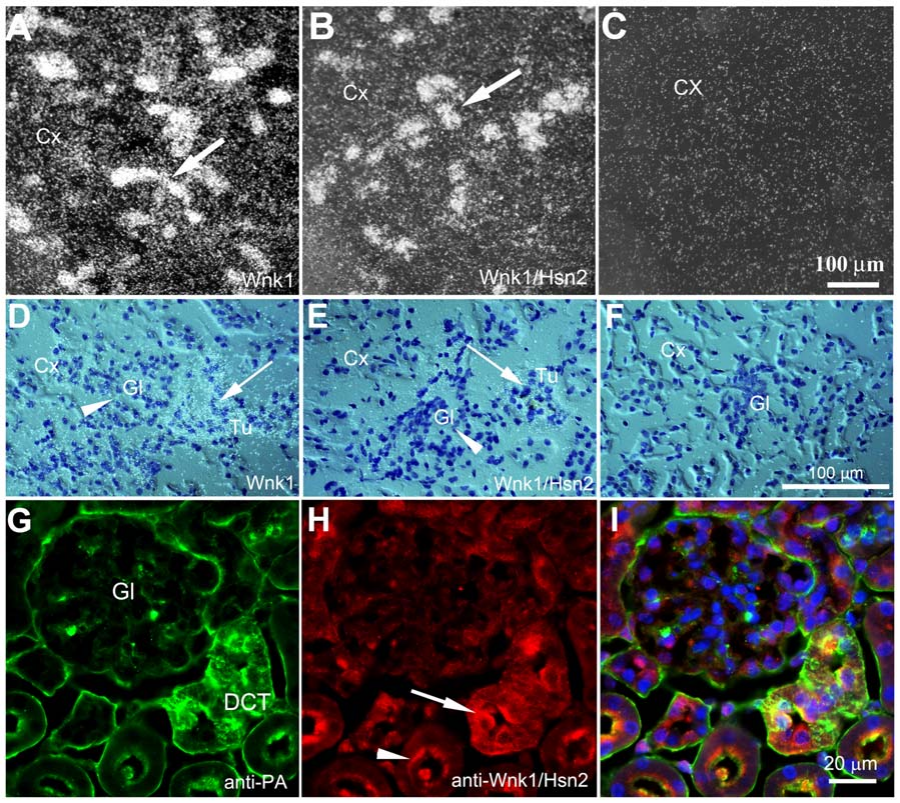
*Wnk1* and *Wnk1/Hsn2* expression in the kidney cortex of adult mice. (A–F) Tissue sections from the kidney cortex were prepared and used in ISH detections with anti-sense *Wnk1*, anti-sense *Wnk1/Hsn2* and sense *Wnk1/Hsn2* (negative control) riboprobes, respectively. Arrows in A and B indicate the increased expression of both *Wnk1* and *Wnk1/Hsn2* in tubules. (D–F) Arrows in D and E indicate, on corresponding emulsion autoradiographies, that the expression of *Wnk1* and *Wnk1/Hsn2* are more predominant in the tubules than the glomeruli (arrowheads in D and E). (G–I) Corresponding sections were co-immunolabeled using anti-parvalbumin (green) and anti-Wnk1/Hsn2 (red) antibodies. The arrow and the arrowhead show cytoplasmic and apical expression of Wnk1/Hsn2 in DCT and convoluted tubules, respectively. Cx  =  cortex; Gl  =  glomerulus; Tu  =  tubule; PA  =  parvalbumin.

**Figure 12:**
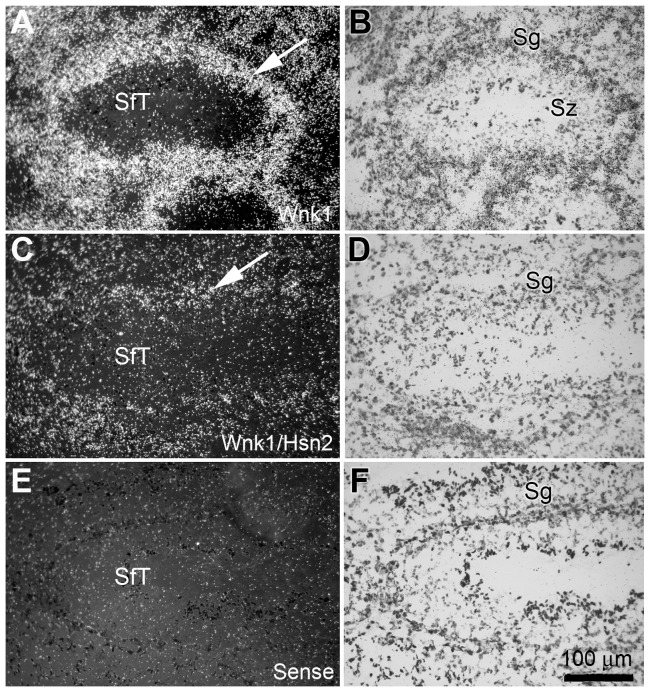
*Wnk1* and *Wnk1/Hsn2* expression in the testis of adult mice. (A, C and E) Tissue sections prepared from mouse testis were used in ISH detections with anti-sense *Wnk1*, anti-sense *Wnk1/Hsn2* and sense *Wnk1/Hsn2* (negative control) riboprobes, respectively. (B, D and F) Corresponding sections were stained with cresyl violet. Arrows in A and C indicate the increased expression of *Wnk1* in the wall of the seminiferous tubule spermatogonia cell layers, in comparision to *Wnk1/Hsn2*. Sc  =  spermatocyte; SfT  =  seminiferous tubule; Sg  =  spermatogonia; Sz  =  spermatozoa.

Surprisingly, some *Wnk1/Hsn2* labeling was also observed in the kidney, thymus, and testis, whereas previous RT-PCR experiments failed to detect any band in two of the tissues: kidney and testis [4]. A more detailed examination of adult mouse kidney sections revealed strong labeling with *Wnk1* or *Wnk1/Hsn2* riboprobes in distal convoluted tubules (DCT) adjacent to glomeruli (Fig. 11A, 11B, 11D and 11E arrows) within the renal cortical region, with little to no labeling detected in the glomeruli (Fig. 11D, 11E arrowheads). Co-immunolabeling of kidney with anti-parvalbumine, which is highly expressed in DCT and anti-Wnk1/Hsn2 antibodies, confirmed the above (Fig. 11G–I). This latter observation is consistent with a previous ISH performed on the adult mouse kidney using a the kidney specific *Wnk1* (*KS-Wnk1*) probe [19]. It also suggests a similar expression pattern for *Wnk1* and *Wnk1/Hsn2*. In line with previous findings [3], [7], [19], [20], we sought to address the expression of a *KS-Wnk1* variant consisting of the Hsn2 exon in the mouse kidney. Using kidney specific exon 4A and Hsn2 primers, RT-PCR experiments revealed *KS-Wnk1* variants consisting of the Hsn2 exon only in kidney. This further confirmed our results on the detection of Hsn2 containing variants in kidney (Fig. S3). Taken together, these results indicate that the *Wnk1/Hsn2* mRNA isoform may be coexpressed with the full length *Wnk1* mRNA in certain non-nervous tissues such as kidney. In testis, the wall of seminiferous tubules (SfT) and the spermatogonia (Sg) cell layer displayed hybridization labeling. A greater intensity of hybridization was detected with the *Wnk1* riboprobe, whereas very low-level labeling was observed with the *Wnk1/Hsn2* riboprobe in the adult mouse (Fig. 12A, 12C, arrows).

## Discussion

To establish a comparative analysis of the expression profiles of *Wnk1* and its sensory associated splice isoform, *Wnk1/Hsn2*, whole body and tissue sections of developing and adult mice were examined using ISH. Recent work on the *WNK1* gene structure suggests the presence of tissue-specific promoters in exons 1 and 4A as well as the presence of multiple splice variants. Therefore, it can be surmised that multiple WNK1 protein variants are expressed and interact with a number of substrates, including other WNK kinases in a tissue-specific fashion. The observation of ubiquitous expression of *Wnk1* at e10.5 in the mouse is consistent with a previous report in which a riboprobe corresponding to exon 6–9 of *Wnk1* showed ubiquitous expression at e10 [16]. Here, we found subtle differences in the signals detected for *Wnk1* and *Wnk1/Hsn2* at e10 and e12.5. Ubiquitous expression suggests that *Wnk1* isoforms may be expressed in the developing cardiovascular system as well as the developing secretory epithelia. Furthermore, it was previously shown that extrarenal *Wnk1* expression is restricted to polarized epithelia playing important roles in Cl^−^ flux [11].

In the brain, *Wnk1* and *Wnk1/Hsn2* mRNAs appeared very early, which suggests a role in brain development. In addition, both riboprobes displayed strong hippocampal signals in the adult mouse indicating some postnatal roles for Wnk1 in hippocampal function. Recently, a Wnk1 and Wnk3 interaction through their C-terminal was shown to be required for Wnk1 activity [21]. In addition, a similar Wnk1 and Wnk3 distribution pattern in the hippocampus may suggest a coexpression in hippocampal neurons [10].

Because of the role of *Wnk1* in HSAN II, we carefully evaluated *Wnk1/Hsn2* isoform expression in cells of the dorsal root. *Wnk1* and *Wnk1/Hsn2* mRNAs were detected in mouse dorsal roots at all ages studied, suggesting that Wnk1 has an important role in development and function of the dorsal root neurons. Weak signals were observed throughout the sections of different tissues using both probes during development. This could be an indication of low *Wnk1* expression in the epithelia of small vessels, capillaries, and arteries as well as in migrating neurons. Although it is difficult to demonstrate the coexpression of *Wnk1/Hsn2* and *Wnk1* specific mRNAs in the same cells, the detection of the same mRNA signal intensity could be an indication of co-expression of multiple *WNK1* isoforms in the same tissue and possibly in the same type of cells. This is plausible, since the WNKs' family members were shown to be coexpressed and to interact with each other *in vitro*. Our results suggest that the *Wnk1/Hsn2* isoform may be expressed in some adult mouse non nervous tissues such as kidney and testis. This was further confirmed by the detection of *Wnk1/Hsn2* variants containing the Hsn2 exon in kidney. This latter finding is contrary to previous observations. The differences could be partly explained by poor synthesis of cDNA in the RT-PCR experiments. Furthermore, detection of some presumably non-specific low molecular weight bands on our Western blots in kidney and liver tissue lysates of adult mouse may also suggest the existence of shorter splice variants containing the Hsn2 domain [4]. Recent work has also suggested that *Wnk1/Hsn2* transcripts are expressed in non-nervous tissue [7].

It will be interesting to explore whether or not *Wnk1* is involved in epithelial development. Our results also confirm that the expression of the *Wnk1/Hsn2* isoform starts at early embryonic stages, inferring the possibility of a role during the development of mouse nervous tissues.

The present study has provided evidence of comparable, but not identical distribution of the two *Wnk1* isoforms in mouse development. Whereas *Wnk1* had a relatively wide distribution pattern, the *Wnk1/Hsn2* isoform showed more restricted mRNA distribution, primarily in neuronal cells of the peripheral ganglia (trigeminal ganglion, dorsal root ganglion, sympathetic pre-aortic ganglion and myenteric plexus's ganglion) as well as hippocampal neurons.

## Supporting Information

Figure S1 **Wnk1/Hsn2 is highly expressed in vessels, neuronal crest cells and proliferative endothelial cells.** (A–C) Illustrate immunohistochemistry of e10.5 mouse sections co-labeled with anti-Wnk1/Hsn2 and anti-nestin antibodies. Arrows in A and B indicate that epithelial areas are strongly labeled with both antibodies. (D–F) Illustrate e10.5 mouse sections which were co-labeled with fluorescein-tomato lectin and anti-Wnk1/Hsn2 to reveal blood vessels and Wnk1/Hsn2, respectively. (G–I) show higher magnification of blood vessels co-labeled in D–F. TL = tomato lectin.
(TIF)

Figure S2 **Wnk1 and Wnk1/Hsn2 expression in the peripheral nervous system of adult mice.** (A–C) Tissue preparations from adult mice cervical DRG were stained with cresyl violet. (D–I) Corresponding sections were used in ISH detections with anti-sense Wnk1, anti-sense Wnk1/Hsn2 and sense Wnk1/Hsn2 (negative control) riboprobes; hybridization signals were detected by emulsion autoradiographies. Arrows in D and E show the expression of Wnk1 and Wnk1/Hsn2 probe in DRG neurons. Arrows in G and H indicate the expression of both Wnk1 and Wnk1/Hsn2 in the neuronal somata. Arrowheads in G and H point to the high labeling of Wnk1/Hsn2 riboprobe in the supporting satellite cells. Dotted circles delineate the boundaries of neuronal somata and their supporting satellite cells.
(TIF)

Figure S3 **Schematic presentation of the position of Wnk1 and Wnk1/Hsn2 probes and two primers designed to amplify the kidney specific Wnk1 isoform (KS-Wnk1).** (A) Presentation of exons 1–10 of Wnk1 cDNA and position of Wnk1 and Wnk1/Hsn2 probes. (B) Presentation of KS-Wnk1 and the position of two primers (1 and 2). (C) RT-PCR of mouse tissues revealed strong expression of KS-Wnk1 only in kidney.
(TIF)
