# Supplementary figures and images for: Comparative Analysis of the Expression Profile of Wnk1 and Wnk1/Hsn2 Splice Variants in Developing and Adult Mouse Tissues
Source: PLoS One. 2013 Feb 25;8(2):e57807. doi: 10.1371/journal.pone.0057807 (PMC3581481; doi:10.1371/journal.pone.0057807)

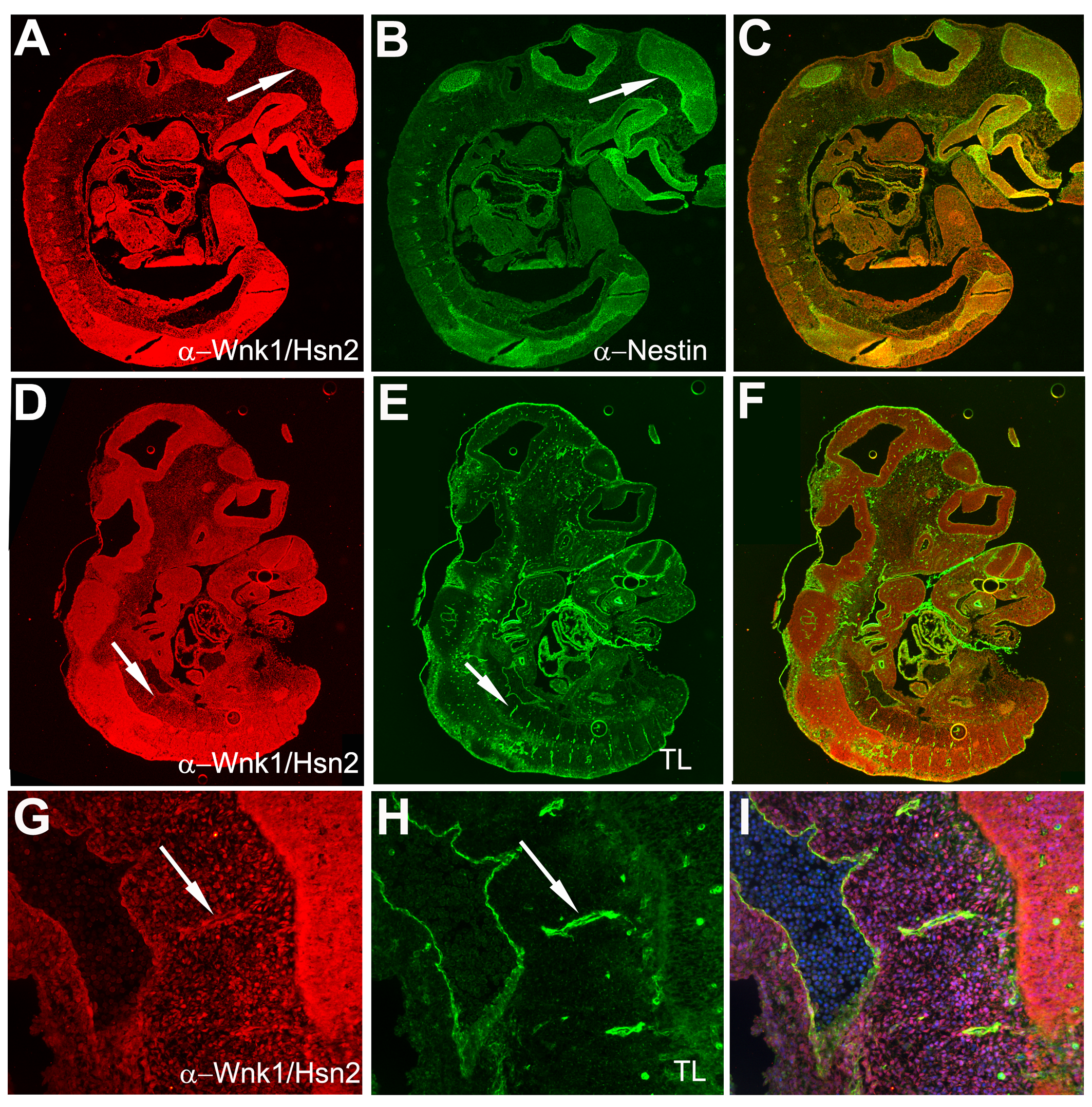

Supplement: Figure S1 — Wnk1/Hsn2 is highly expressed in vessels, neuronal crest cells and proliferative endothelial cells. (A–C) Illustrate immunohistochemistry of e10.5 mouse sections co-labeled with anti-Wnk1/Hsn2 and anti-nestin antibodies. Arrows in A and B indicate that epithelial areas are strongly labeled with both antibodies. (D–F) Illustrate e10.5 mouse sections which were co-labeled with fluorescein-tomato lectin and anti-Wnk1/Hsn2 to reveal blood vessels and Wnk1/Hsn2, respectively. (G–I) show higher magnification of blood vessels co-labeled in D–F. TL = tomato lectin. (TIF) [file pone.0057807.s001.tif]

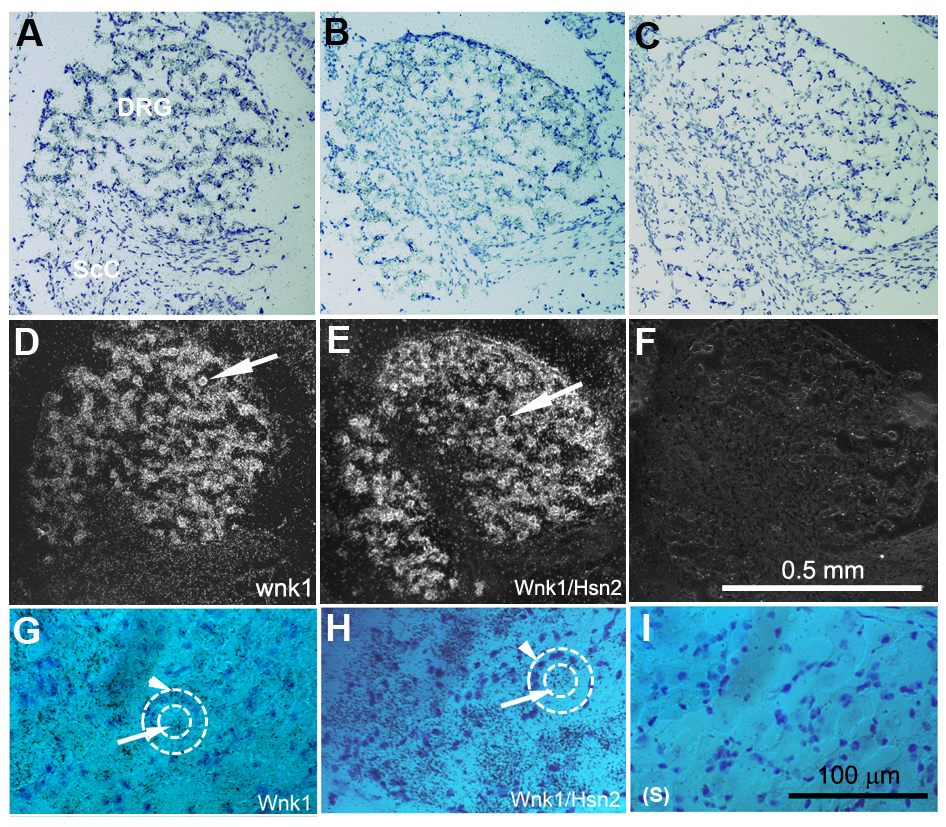

Supplement: Figure S2 — Wnk1 and Wnk1/Hsn2 expression in the peripheral nervous system of adult mice. (A–C) Tissue preparations from adult mice cervical DRG were stained with cresyl violet. (D–I) Corresponding sections were used in ISH detections with anti-sense Wnk1, anti-sense Wnk1/Hsn2 and sense Wnk1/Hsn2 (negative control) riboprobes; hybridization signals were detected by emulsion autoradiographies. Arrows in D and E show the expression of Wnk1 and Wnk1/Hsn2 probe in DRG neurons. Arrows in G and H indicate the expression of both Wnk1 and Wnk1/Hsn2 in the neuronal somata. Arrowheads in G and H point to the high labeling of Wnk1/Hsn2 riboprobe in the supporting satellite cells. Dotted circles delineate the boundaries of neuronal somata and their supporting satellite cells. (TIF) [file pone.0057807.s002.tif]

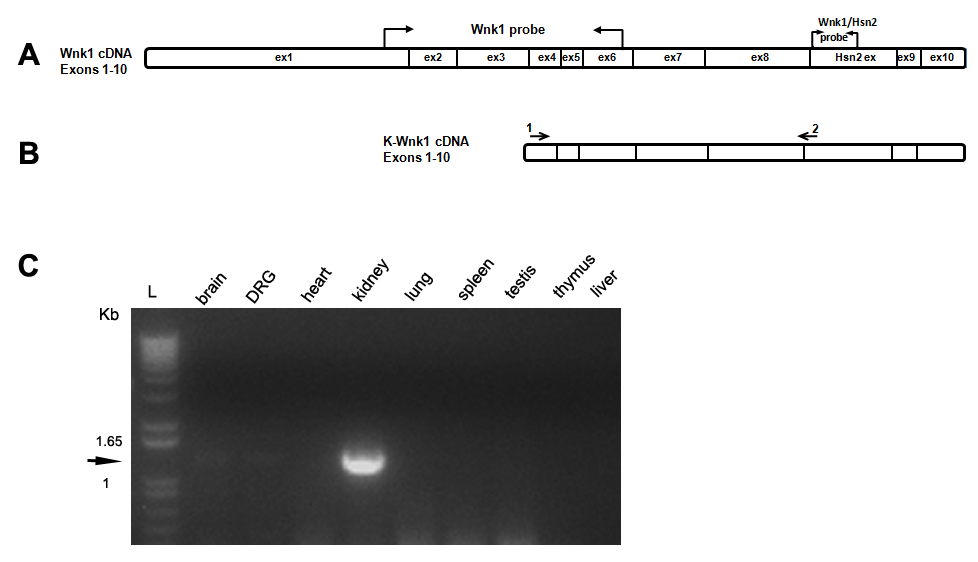

Supplement: Figure S3 — Schematic presentation of the position of Wnk1 and Wnk1/Hsn2 probes and two primers designed to amplify the kidney specific Wnk1 isoform (KS-Wnk1). (A) Presentation of exons 1–10 of Wnk1 cDNA and position of Wnk1 and Wnk1/Hsn2 probes. (B) Presentation of KS-Wnk1 and the position of two primers (1 and 2). (C) RT-PCR of mouse tissues revealed strong expression of KS-Wnk1 only in kidney. (TIF) [file pone.0057807.s003.tif]
